# Risk factors for myopia in a discordant monozygotic twin study

**DOI:** 10.1111/opo.12246

**Published:** 2015-09-17

**Authors:** Rishi Ramessur, Katie M. Williams, Christopher J. Hammond

**Affiliations:** ^1^ Department of Twin ResearchKings College LondonLondonUK; ^2^ Department of MedicineUniversity of OxfordOxfordUK

**Keywords:** epigenetics, myopia, refractive error, risk factors

## Abstract

**Purpose:**

Monozygotic (MZ) twin pairs discordant for disease allow careful examination of environmental factors whilst controlling for genetic variation. The purpose of this study was to examine differences in environmental risk factors in MZ twins discordant for myopia.

**Methods:**

Sixty four MZ twin pairs discordant for refractive error were interviewed. Discordant twins were selected from 1326 MZ twin pairs from the TwinsUK adult twin registry with non‐cycloplegic autorefraction. Discordancy was defined as ≥2 Dioptres (D) difference in spherical equivalent (SphE) and discordant for class of refractive error.

In a 35‐item telephone questionnaire twins were separately asked (and scored) about the risk factors urban/rural residence, occupational status and highest educational level. They responded with more (1), less (−1) or the same (0) as their twin on time spent outside, playing outdoor sport, and on close work aged <16 and 16–25 years. The lower SphE twin's score was subtracted from the higher SphE twin's score, and mean values of the difference calculated for each variable.

**Results:**

Sixty four twin pairs were included (mean age 56, range 30–79 years; mean difference in refraction 3.35 D, S.D. 1.55 D, median difference 2.78 D). Within discordant MZ twin pairs, the more myopic twin was associated with having a higher occupational status (mean score between 16 and 25 years −0.11; 95% CI −0.19 to −0.04; mean score aged >25 years −0.23, 95% CI −0.28 to −0.17), being resident in urban area (mean score −0.26; 95% CI −0.33 to −0.18) and performing more close work (mean score <16 years −0.11; 95% CI −0.18 to −0.05; mean score aged 16–25 years −0.17, 95% CI −0.24 to −0.10) than their twin. The twins who spent more time outdoors (mean score <16 years 0.09; 95% CI 0.03–0.15; mean score aged 16–25 years 0.28, 95% CI 0.15–0.41) or performed more outdoors sports (mean score <16 years 0.13; 95% CI 0.04–0.21; mean score aged 16–25 years 0.23, 95% CI 0.10–0.36) were less likely to be myopic than their twin.

**Conclusions:**

This study has confirmed known environmental risk factors for myopia. These data will allow selection of discordant twins for epigenetic analysis to advance knowledge of mechanisms of refractive error development.

## Introduction

Myopia affects over a third of adults in the UK,^1^ and is a frequent cause of visual impairment and morbidity worldwide.^2^, ^3^ Myopia confers increased risk of sight‐threatening ocular pathology, particularly amongst highly myopic individuals [Spherical Equivalent ≤ −6 Dioptres (D)] who account for 2–4% of the population across Europe, Australia and United States.^4^, ^5^, ^6^ Myopia's significant burden also includes cost of correction^7^, and uncorrected vision is associated with increased risk of falls (particularly in the elderly)^8^, ^9^, ^10^ and other societal costs.^11^ Recent trends in the prevalence of myopia (61.2% in Taiwanese 15 year olds in 1983 to 81% in 2000) and rising levels of high myopia (over 20% in young adults across Asian populations)^12^, ^13^, ^14^, ^15^, are likely to pose a future burden on public health and demonstrate important environmental drivers of myopia.

Studies on school children have reported associations between greater levels of outdoor (but not indoor) sports and activity, and reduced prevalence of myopia‐ independent of levels of near work.^16^, ^17^, ^18^, ^19^ The role of near work in myopia is less clear. Children who spend longer reading for pleasure and who read from closer distances have been shown to be more myopic.^20^, ^21^ However, the Orinda Longitudinal Study for Myopia (OLSM) showed that the likely effects of typical differences in levels of near work between children are small.^22^ In a follow‐on study, myopic children spent more time on close work activities than emmetropes at the time of onset of myopia and in four of the 5 years following onset, but there was no difference prior to the onset of myopia, highlighting potential reverse causality.^23^ Living in urban areas, having a higher IQ and greater level of educational attainment have also been noted as risk factors.^24^, ^25^, ^26^ The mechanisms by which these factors influence refractive error in humans are largely unknown.

Classical twin studies suggest that refractive error is highly heritable (over 80%) across different ages and populations.^27^, ^28^ Axial length and myopia prevalence increased dose‐dependently with 0, 1 or 2 myopic parents in the Sydney Myopia Study.^29^ The severity of myopia in children correlated with the severity of myopia in either parent. Other studies report similar findings.^20^, ^30^ In addition the OLSM showed that in non‐myopic children, those with myopic parents had longer axial lengths than children without myopic parents, even before myopia onset.^30^ Genome‐wide association studies have identified multiple loci associated with myopia.^31^, ^32^ Environmental factors might influence refractive error through gene‐environment interaction, as has been suggested for education^33^, or by altering epigenetic regulation of gene expression.

The discordant identical twin model is a powerful tool in assessing the impact of environmental modifiers on a trait, since monozygotic twins share the same genotype, eliminating genetic variation as a cause of discordance, and also share many early life factors. The discordant identical twin model may also be the perfect design to study epigenetics. No study to date has examined environmental influences for refractive error using this model and current literature is limited to individual case reports of discordant twins.^34^, ^35^ We aimed to determine whether monozygotic twins discordant for refractive error had differing environments during adolescence and early adulthood. Using open ‘qualitative’ research, we also set out to explore any potential theories, ideas and perceptions about myopia within discordant twin pairs.

## Methods

The TwinsUK cohort is a registry of British twins based at St Thomas' Hospital in London and recruited over the past 22 years with a mean age of 51 years (range 18–80), who have participated in genetic and other studies of ageing (including eye disease).^36^ Subjects volunteered to be on the registry following media campaigns and were invited to the hospital for phenotyping (venepuncture and measurements) according to the tenets of the Declaration of Helsinki and with local Research Ethics Committee approvals. Zygosity was determined by standardised questionnaire.^37^ DNA short‐tandem repeat fingerprinting or genome‐wide association data were used to confirm true zygosity if there was any doubt from the twins or the investigator, or the questionnaire did not definitively categorise subjects as either monozygotic or dizygotic. The TwinsUK cohort is largely female, in part for historic reasons (initial recruitment was female‐only) and subsequently because of female volunteer bias, common to all twin registries.

Refractive error, recorded as mean spherical equivalent (SphE) of both eyes from non‐cycloplegic autorefraction (ARM‐10; www.takagi-seiko.co.jp/en/), has been measured since 1998 for over 6000 twins. Where refraction was performed on more than one occasion, the earliest obtained refraction was used. We have previously reported a classical heritability study from over 2000 twin pairs, which showed that monozygotic twin pairs are highly correlated for SphE (*r* = 0.8).^38^ Exclusion criteria included subjects with previous cataract or refractive surgery, or other conditions which might alter refraction, or where data from both twins of a pair were unavailable.

The mean difference in SphE between MZ pairs was 0.92 D (SD 1.07 D). MZ twin pairs were defined as discordant for refractive error, if they had a ≥2 D difference in SphE and were discordant for class of refractive error (high/moderate/low myopia, emmetropia, hyperopia). We defined hyperopia as refractive error ≥0.50 D (low 0.50–2.9 D, moderate 3.0–5.9 D, and high ≥6.0 D) and myopia as ≤−0.50 D (high ≤−6.0 D, moderate −5.9 D to −3.0 D, and low −2.9 D to −0.50 D).

In a 35‐item telephone questionnaire (Appendix 1) discordant twin pairs were separately asked to rate if they spent more, less, or the same amount of time on three activities, associated with myopia, when compared to their twin (scored respectively as 1, −1 or 0). The three activities questioned were time spent outside, playing outdoor sport, and close work (defined as the cumulative time spent on studying, reading, sewing/knitting and any other type of close work). This question was posed for two retrospective time points: aged <16 years, and aged 16–25 years. Subjects were asked about their own age when leaving full‐time education and qualifications gained; a score of 0–5 was calculated depending on their answers (Table S1). Similarly, job status was rated on a scale from 0 to 5, modified from the Registrar General's social class classification (Table S2). The degree of urbanisation was scored as −1 for urban area, 0 for suburban and 1 for rural residence, with an average taken if participants moved to a different category of residence between the ages of 16 and 25.

**Figure 1:**
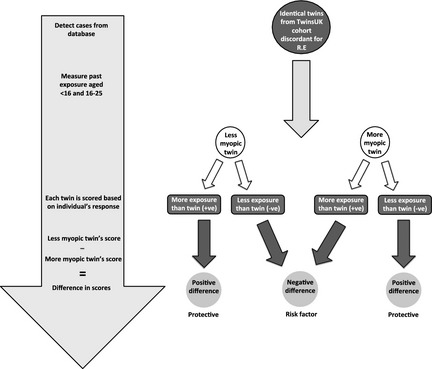
The discordant twin model is a case control study in which monozygotic twins with discordant refractive error act as perfectly matched genetic controls to examine environmental differences. RE, Refractive error.

Scores were standardised to a common scale of −2 to +2 for each variable (Appendix 2), a positive score reflecting greater reported exposure and a negative score reflecting less reported exposure. The standardised score for each variable of the twin with lower SphE was subtracted from the score for the twin with higher SphE. Thus one would expect that for a protective environmental variable, the less myopic of the pair would report more exposure (+1) compared to the more myopic twin (−1), thus yielding a positive difference (+1 minus −1 = +2). Conversely, a risk factor for myopia would be expected to yield a negative difference (see *Figure* 1). For example, to score <0 for current job, the twin with lower SphE had higher occupational status at time of questioning. To score <0 for education, the twin with lower SphE was older when leaving full‐time education or achieved a higher level of educational qualifications. Mean values and 95% confidence intervals of the differences were calculated.

In order to determine whether there might be confirmation bias (affected twins more likely to confirm their beliefs about risk factors), two open questions (one at the beginning and one at the end, Appendix 1) allowed subjects to explore any theories or ideas regarding their discordancy. Theoretical framework was based on grounded theory (building theories from the data) and exploring sample characteristics, such as prevalence of preconceptions about myopia risk factors. Key themes regarding their theories and ideas were obtained from the notes of the interviews and categorised according to their common properties. Although interviews were not audio recorded, subjects' responses were summarized back to them to clarify the accuracy of the notes. The total number of subjects suggesting each theory was separated by number of responses from higher and from lower SphE twins, and a chi squared test was used to assess whether there was a significant difference between the groups.

## Results

**Figure 2:**
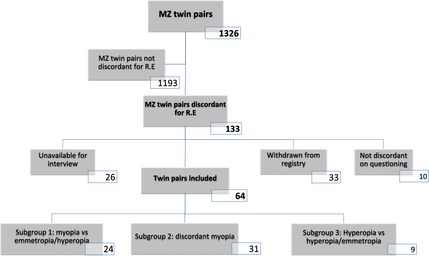
Subjects included and reasons for exclusion**.**

Autorefraction was available for both members of 1326 same‐sex monozygotic twin pairs. 133 twin pairs were classified as discordant for refractive error (126F, 7M; mean age 57, range 30–89 years; mean difference in refraction 3.32 D, S.D. 1.59 D), using the criteria of >2 D difference and being in a different category of refractive error to their twin. Of these, 33 pairs had withdrawn from the TwinsUK registry due to death (8 pairs), voluntary withdrawal (12 pairs), disability (3 pairs), or relocation (10 pairs). On questioning, 10 were found not to be discordant for refractive error (due to unrecorded cataract surgery, excimer laser or glaucoma surgery altering refraction, or data entry error). In 26 of the remaining twin pairs, one (9 pairs) or both (17 pairs) twins were unavailable for interview (uncontactable, or declined to participate). This resulted in 64 pairs of MZ twins discordant for refractive error who could be interviewed (mean age 56, range 30–79 years, mean difference between pairs 3.35 D, S.D. 1.55 D, median difference 2.78 D), and 69 twin pairs not interviewed (mean age 59, range 30–89 years, mean difference between pairs 3.29 D, S.D. 1.64 D, median difference 2.75 D). The twin pairs included were categorised into three subgroups: Subgroup 1 (24 pairs), in which one twin was myopic and the other twin was not (mean age 58, range 32–78 years, mean difference between pairs 3.65 D, S.D. 2.02 D, median difference 2.81 D); Subgroup 2 (31 pairs)‐ both twins were myopic but discordant for class of myopia (mean age 55, range 30–79 years, mean difference between pairs 3.24 D, S.D. 1.27 D, median difference 2.75 D); and subgroup 3 (9 pairs), in which one twin was hyperopic and the other twin either emmetropic or fell into a different class (low/medium/high) of hyperopia (mean age 59, range 52–71 years, mean difference between pairs 2.91 D, S.D. 0.33 D, median difference 2.88 D). *Figure* 2 summarises the subjects included. Differences in refractive error were calculated by subtracting the lower of the two refractive errors from the higher of the two.

Concordant and discordant twin pairs were broadly comparable in terms of distribution of risk factors: 25% and 24% achieved higher education respectively, and 30% in both groups were in the most affluent class as defined by the Index of Multiple Deprivation (based on UK postcodes).

**Figure 3:**
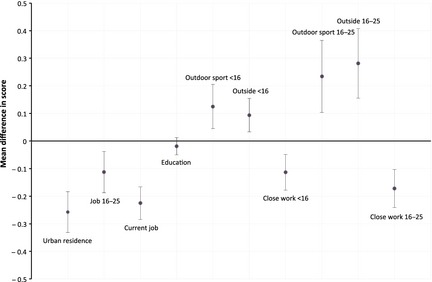
Mean differences in self‐ and twin‐rated scores between higher SphE and lower SphE twins for environmental variables in all subgroups of monozygotic twin pairs discordant for refractive error.

Overall, the more myopic/less hyperopic twins self‐reported higher occupational status (mean score between 16 and 25 years −0.11; 95% CI −0.19 to −0.04; mean score aged >25 years −0.23, 95% CI −0.28 to −0.17), more close work (mean score <16 years −0.11; 95% CI −0.18 to −0.05; mean score aged 16–25 years −0.17, 95% CI −0.24 to −0.10), and were more likely to live in urban areas (mean score −0.26; 95% CI −0.33 to −0.18) than their twin. They also spent less time outside (mean score <16 years 0.09; 95% CI 0.03–0.15; mean score aged 16–25 years 0.28, 95% CI 0.15–0.41) and did less outdoor sport (mean score <16 years 0.13; 95% CI 0.04–0.21; mean score aged 16–25 years 0.23, 95% CI 0.10–0.36) (*Figure* 3).

Analysis of the myopic discordant twins (subgroups 1 and 2, 55 pairs of twins) showed similar findings to the overall analysis (Figure S1). Of the two subgroups, the differences were most pronounced in the group where one twin was myopic and the other was not (Figure S2). There were no obvious differences within subgroup 3 (the hyperopic‐discordant twins), reflecting the small sample size (9 pairs).

At the start of the interview, after discordance was confirmed, subjects' understanding of the potential reasons for their discordance was explored in qualitative questioning. *Table* 1 lists possible reasons for discordance given by 128 twins. 28 twins were aware of close work as a possible reason for the discordance in refractive error. 26 twins attributed discordance to occupational status, 23 to where they lived in adulthood, 17 to other illnesses or medication 16 twins to heredity, 10 to prematurity, 10 to diet or lifestyle and 9 to outdoor activity. 9 out of 18 subjects interviewed from subgroup 3 (the hyperopic‐discordant twins) considered measles infection at a young age as a theory for their discordance. There was no significant difference between the prior knowledge of the higher SphE twin compared to the lower SphE twin for any of the risk factors listed (see *Table* 1, *p*‐values).

**Table 1:** Qualitative questioning about the possible reasons for discordance (prior to questionnaire), divided into total number of twins, number of twins with higher SphE and with lower SphE suggesting possible reasons

Theory	*N* (Total)	*N* (Higher SphE)	*N* (Lower SphE)	*p*‐value
Close work	28	12	16	0.45
Job vs raising family before 25	26	14	12	0.69
Residence	23	10	13	0.53
Association with other illnesses/medication (e.g. measles, depression, asthma medication)	17	9	8	0.81
Heredity	16	6	10	0.32
Prematurity/lower birth weight	10	5	5	1
Diet/life style	10	6	4	0.53
Outdoor exposure/night work and poor light	9	3	6	0.32
Injury	5	2	3	0.65
Mirror image twins (theory that phenotypes are opposite for certain traits)	4	3	1	0.32
Other (contact lenes, hair occluding eyes, birth trauma, eye deformity at birth, pregnancy‐associated myopia, eye exercising, wealth)	11	5	6	0.76

## Discussion

Our study of discordant middle‐aged monozygotic twins confirms key risk factors for myopia and suggests differences in lifestyles before 25 years of age have a sustained effect, reflected in twin subjects' refraction over 30 years later. This raises the possibility that there may be long‐lasting epigenetic differences between discordant MZ twins that could improve our understanding of how the environment alters ocular growth and homeostasis.

The strength of associations for discordancy in close work, time spent outside and outdoor sport were stronger between ages 16 and 25 than they were before age 16. We used these age brackets because identical twins likely spend most of their childhood, until age 16, engaging in similar activities. After 16, their lifestyles may diverge. This greater divergence may explain the stronger associations in the older age group, although ease of recall for more recent events could also account for this. While much of myopia is assumed to be ‘school‐onset’, a considerable proportion (42.3%) of myopes in our cohort first wore spectacles after the age of 16 years.^39^ Most studies regarding environmental influences on myopia have been performed in younger populations,^2^, ^16^ although there is significant myopia incidence and progression in adulthood.^39^, ^40^ Indeed, some of our myopic subjects would have been misclassified if studied at age 18 or below.

Near work and educational attainment have been shown to be independent risk factors in multivariate analysis^22^ but there may also be a shared genetic contribution to educational attainment and myopia development.^41^ Obviously the MZ twins have identical genotypes, so the myopia‐discordant twins showing discordance for educational level implies that it is a separate risk factor, independent of genetic effects.

Qualitative questioning revealed that twin subjects were most aware of the ‘close work’ theory of myopia development; even so, only 28 (22%) of the 128 twins reported close work as a possible reason for their discordant myopia status. There was little knowledge about the protective effects of outdoor activity/light, with only 8% suggesting it as a risk factor or reason for discordance. Interestingly 16/128 subjects appeared poorly informed as they suggested genetics as a cause of discordancy, despite being monozygotic twins and participating in twin research with regular newsletters about the twin model, although it is possible they were aware of the epigenetic research in TwinsUK. The lack of prior knowledge of myopia risk factors suggests little confirmation bias in this discordant MZ study.

In terms of hypotheses for myopia risk factors suggested in the qualitative arm of the study, a relatively common suggestion was that the lower birth weight twin might be at a greater risk of developing myopia. Age‐adjusted regression analysis on over 4000 twins revealed no significant association between birthweight and refraction within the overall TwinsUK cohort (*p* = 0.39) or within MZ twins only (*p* = 0.062).

This study has found a lack of awareness of the protective effects of outdoor activity, which have been widely reported in epidemiological studies of myopia^16^, ^17^, ^18^, ^19^, so we would recommend a public health policy to promote the positive effects of outdoor activity to reduce the rising trend of myopia.

The predominantly female sample of British subjects may not be generalisable to both genders and other population groups.^42^ Subjects volunteered for the TwinsUK registry unaware of specific myopia studies, autorefraction was just one of many measurements taken as part of a broad TwinsUK study, thus reducing ascertainment bias. Considering the sample age range, the lack of cycloplegia in autorefraction is unlikely to confound this study.^43^

The case‐control nature of the study lacks the power to dissociate between outcomes and predictors. It is feasible that individuals with greater refractive errors are less inclined to engage in outdoor activity due to spectacle wear, although multiple studies have found close work to be a risk factor independent of outdoor activity.^16^, ^18^

The subjective nature of parts of the questionnaire may lead to recall bias, particularly given the level of awareness of the association of close work with refractive error. The nature of the questionnaire relied on subjects' ability to recall, often over decades. This was partly addressed within the study by interviewing the twins separately, thus increasing the confidence in recall. Conversely, the ability of the study to detect associations given the length of recall shows how powerful a tool the discordant twin model is, with implications for future studies.

Despite the retrospective recall nature of this study, the discordant monozygotic twin model has confirmed known environmental risk factors in participants lacking prior knowledge of potential modifiers of refractive error. We have shown that the strongest effects were seen comparing the twin pairs where one was myopic and the other emmetropic or hyperopic, highlighting that comparing affected myopic subjects against ‘unaffected’ may be more powerful than comparing within a group of subjects with differing degrees of myopia. Recent studies highlight the potential for epigenetic events, such as alterations in gene expression by DNA methylation, to explain discordancy in monozygotic twins.^44^ The discordant monozygotic twin model is a powerful tool and a follow‐up study will look for differentially methylated regions in monozygotic twins identified in this study as discordant for myopia.

## Disclosure

The authors report no conflicts of interest and have no proprietary interest in any of the materials mentioned in this article.

## Supporting information


**Figure S1.** Supplementary Figure 1. Mean differences in self‐ and twin‐rated scores between higher SphE and lower SphE twins for subgroups 1 + 2 (myopia vs emmetropia/hyperopia and discordant myopia)

**Figure S2.** Supplementary Figure 2. Mean differences in self‐ and twin‐rated scores between higher SphE and lower SphE twins for subgroup 1 (myopia vs emmetropia/hyperopia)

**Table S1.** Supplementary table 1: Scoring criteria for educational status based on participants' questionnaire responses

**Table S2.** Supplementary table 2: Scoring criteria for occupational status based on participants' questionnaire responses.

## Appendix 1 TwinsUK telephone questionnaire conducted between April and September 2014

1. According to our records, your twin's eye‐sight is not quite the same as yours,
  (a). Is this something you were aware of?
  YES NO
  (b). Do you have any theories/ideas as to why your eye‐sights are different?
2. If you have your prescription handy, could you tell the “Sph” values for the right and the left eye.
  RIGHT: LEFT:
3. (a) Have you ever had any problems with your eyes/operations
  YES NO
  (b) If yes, what problems/operations have you had? Problems/operations:
4. (a) Have you ever needed glasses or contact lenses, and
  YES NO
  (b) If so, how old were you when you first needed them?
  Age:
5. (a) Have you ever been married?
  YES NO
  (b) At what age did you get married (age of first marriage)?
  Age:
6. (a) Do you have any children?
  YES NO
  (b) How many of your children have never needed glasses/contact lenses?
  Number of children:
  (c) How many of your children are short‐sighted Number of children:
  (d) How many of your children are long‐sighted
  Number of children:
7. (a) What is your occupation (if retired, what job/jobs did you have for the majority of your working life)?
  Occupation:
  (b) And your partner?
  Partner's occupation:
8. (a) How old were you when you left full‐time education?
  Age:
  (b) Did you go to college/university?
  YES NO
  (c) If yes, did you do a post‐graduate degree?
  YES NO
  (d) What is your highest educational/ school qualification obtained?
  GCSE O‐LEVEL A‐LEVEL DEGREE
  OTHER:
  NONE OF THE ABOVE
9. Before 16, compared to your twin, would you say you played more, less or the same amount of outdoor sport?
  MORE LESS SAME
10. Before 16, compared to your twin, would you say you spent more, less or the same amount of time outside?
  MORE LESS SAME
11. Before 16, compared to your twin, did you spend more, less or the same amount of time on school work?
  MORE LESS SAME
12. Before 16, compared to your twin, did you spend more, less or the same amount of time reading for leisure?
  MORE LESS SAME
13. Before 16, compared to your twin, did you spend more, less or the same amount of time activities such as sewing or knitting?
  MORE LESS SAME
14. Before 16, compared to your twin, did you spend more, less or the same amount of time on any other type of close/near work?
  MORE LESS SAME
15. Between the ages of 16 and 25, in what sort of area did you live?
  URBAN SUBURBAN RURAL
16. Between the ages of 16 and 25, compared to your twin, would you say you played more, less or the same amount of outdoor sport?
  MORE LESS SAME
17. Between the ages of 16 and 25, compared to your twin, would you say you spent more, less or the same amount of time outside?
  MORE LESS SAME
18. Between the ages of 16 and 25, compared to your twin, did you spend more, less or the same amount of time on studying?
  MORE LESS SAME
19. Between the ages of 16 and 25, compared to your twin, did you spend more, less or the same amount of time reading for leisure?
  MORE LESS SAME
20. Between the ages of 16 and 25, compared to your twin, did you spend more, less or the same amount of time activities such as sewing or knitting?
  MORE LESS SAME
21. Between the ages of 16 and 25, compared to your twin, did you spend more, less or the same amount of time on any other type of close/near work?
  MORE LESS SAME
22. (a) Between the ages of 16 and 25, what jobs did you have?
  Occupation:
  (b) And your partner (if you were married at the time)?
  Partner's occupation:
23. Before the age of 25, was there any major difference in your life compared to your twin's?

## Appendix 2 Formula for normalising scores to common scale (−2 to +2). *y* denotes the normalised score, *x* is the original score. *A* and *B* are the minimum and maximum scores on the original scale, respectively

$$y=-2+\frac{4(x-A)}{\left(B-A\right)}$$
