# Supplementary figures and images for: Risk factors for myopia in a discordant monozygotic twin study
Source: Ophthalmic Physiol Opt. 2015 Sep 17;35(6):643–51. doi: 10.1111/opo.12246 (PMC4832275; doi:10.1111/opo.12246)

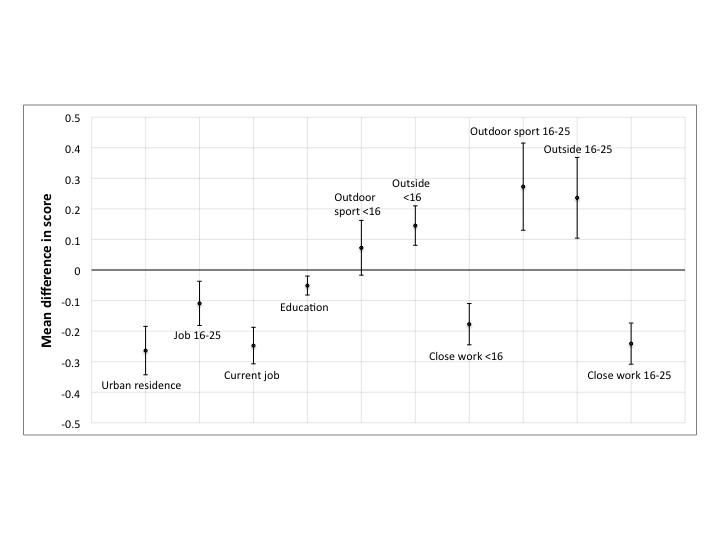

Supplement: Supplementary file 1 — Figure S1. Supplementary Figure 1. Mean differences in self- and twin-rated scores between higher SphE and lower SphE twins for subgroups 1 + 2 (myopia vs emmetropia/hyperopia and discordant myopia) [file 44402_2015_3506010_MOESM1_ESM.tif]

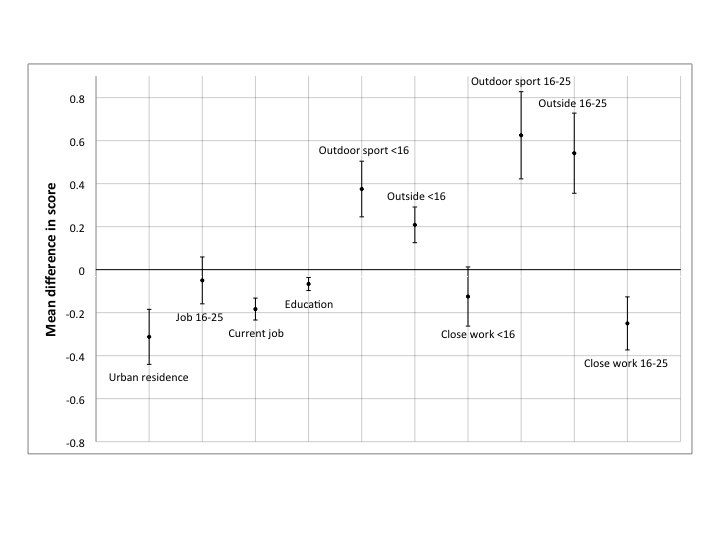

Supplement: Supplementary file 2 — Figure S2. Supplementary Figure 2. Mean differences in self- and twin-rated scores between higher SphE and lower SphE twins for subgroup 1 (myopia vs emmetropia/hyperopia) [file 44402_2015_3506010_MOESM2_ESM.tif]

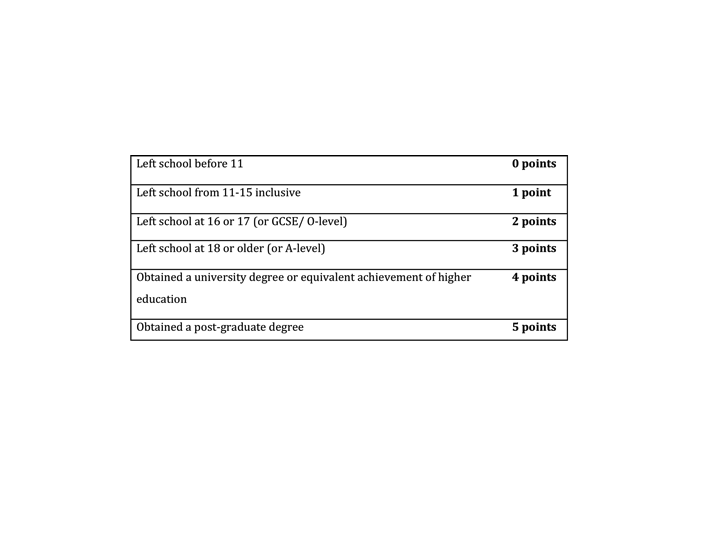

Supplement: Supplementary file 3 — Table S1. Supplementary table 1: Scoring criteria for educational status based on participants' questionnaire responses [file 44402_2015_3506010_MOESM3_ESM.tif]

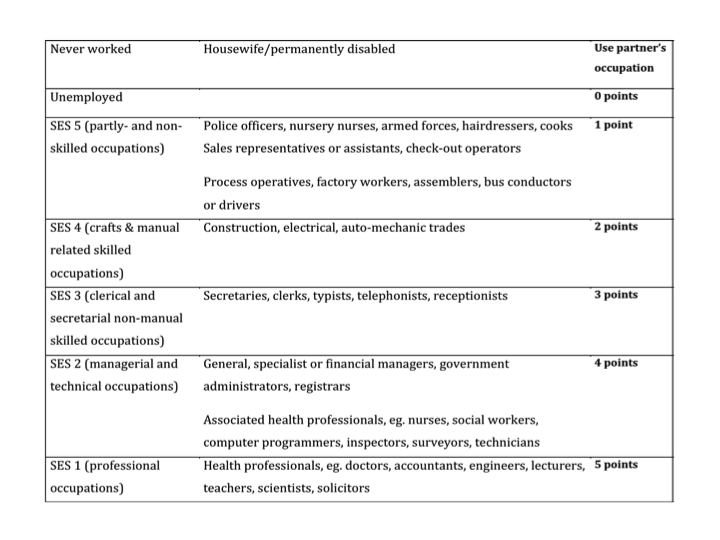

Supplement: Supplementary file 4 — Table S2. Supplementary table 2: Scoring criteria for occupational status based on participants' questionnaire responses. [file 44402_2015_3506010_MOESM4_ESM.tif]
